# Mistaken Notions of Hospitality, and Fancied Security

**Published:** 1888-09

**Authors:** 


### MISTAKEN NOTIONS OF HOSPITALITY, AND FANCIED SECURITY.

Atlanta, by vote of her Council, has, as the Medical and Surgical Journal expresses it, "thrown open her doors to the refugees" from the yellow fever districts of Florida, and invites them to come and dwell in the Gate City. The Medical and Surgical

Journal, commenting upon the act, approves it, and says that Atlanta is yellow fever proof,—that yellow fever has never appeared there, and that no city of equal altitude was ever scourged by the pest; that cities where yellow fever has prevailed, are situated on the coast, or on some river.

We are somewhat surprised at our brother's lack of information on the subject; and at his confidence in the security of Atlanta by virtue of her altitude. Holly Springs, Miss., did the same unwise thing in 1878, with reference to the refugees from New Orleans, boasting the same exemptions which our friends of Atlanta boast they possess; and the refugees flocked to the hospitable little city. In a very short time yellow fever broke out, and for weeks the fair city of hills—and of exalted "altitude"—and which is, moreover, neither by the sea, nor on a water course,—was a scene of mourning and desolation. There the brave doctors—from all sections—notably from Texas, battled with the pestilence; there the gallant Manning fell;—in brief, Holly Springs paid the penalty of her misplaced sympathy, and taught a lesson, which should not be disregarded. Yellow fever is a most insidious foe and is no respector of persons, nor of "altitude"; it has been known to occur (in a refugee) on the summit of Lookout mountain; in the clean, as well as in the filthy cities.

In light of these established facts, Atlanta, nor any other city, should be permitted to harbor the refugees from a fever stricken city; such persons should be detained ten days in camp of inspection, under supervision of the United States government officers; then they might, after disinfection, proceed to enjoy the hospitality of Atlanta or other city; but the safety of certainly 70,000 persons, perhaps many more, should not be jeopardized through any such morbid sentimentality as that which apparently prompts Atlanta, and prompted Holly Springs—*to invite* a pestilence—along with their guests. It is to be hoped that Dr. Hamilton will have "a say" as to refugees flocking to Atlanta—if the city whence they fled is infected. If these persons are panic stricken, and have fled before exposure to possible infection, there can be no objection to their going to Atlanta or elsewhere; and to refuse them entrance would be the refinement of cruelty. In such event there is no occasion to boast—they could go anywhere. Dr. Hamilton should know whether they carry infection with them, and doubtless will.

